# Omaveloxolone and TX63682 are hepatoprotective in the STAM mouse model of nonalcoholic steatohepatitis

**DOI:** 10.1002/jbt.22526

**Published:** 2020-05-14

**Authors:** Scott A. Reisman, Deborah A. Ferguson, Chun‐Yue I. Lee, Joel W. Proksch

**Affiliations:** ^1^ Reata Pharmaceuticals, Inc Irving Texas

**Keywords:** hepatoprotection, mitochondria, NASH, Nrf2, omaveloxolone

## Abstract

Omaveloxolone is a potent activator of Nrf2, a master transcriptional regulator of a multitude of cytoprotective functions, including antioxidative, anti‐inflammatory, and mitochondrial bioenergetic effects. Some of the most potent known effects of Nrf2 involve hepatoprotective functions. The purpose of this study was to evaluate the effects of omaveloxolone and TX63682, a closely related structural analog with similar oral bioavailability, in the STAM mouse model of nonalcoholic steatohepatitis (NASH). C57Bl/6 mice received a single subcutaneous injection of streptozotocin two days after birth and were fed a high‐fat diet from 4 to 9 weeks of age. Omaveloxolone and TX63682 were orally administered at doses of 1, 3, and 10 mg/kg/d from 6 to 9 weeks of age. Consistent with the beneficial effects of Nrf2 on hepatoprotection and improved lipid handling, both omaveloxolone and TX63682 decreased hepatic fat deposition, hepatocellular ballooning, inflammatory cell infiltration, and collagen deposition. Omaveloxolone and TX63682 also improved blood glucose control, as evidenced by reductions in nonfasting blood glucose and glycated hemoglobin A_1C_ concentrations. Reductions in liver and serum triglycerides with omaveloxolone and TX63682 treatment were also observed. Both omaveloxolone and TX63682 decreased leptin and increased adiponectin in serum, which is consistent with the anti‐inflammatory and antifibrotic effects observed in the liver. These results were associated with significant induction of Nrf2 target gene expression in the liver, including NAD(P)H:quinone oxidoreductase 1, sulfiredoxin 1, and ferritin heavy chain 1. Overall, these data suggest that omaveloxolone and related Nrf2 activators may be useful for the treatment of NASH.

## 1 INTRODUCTION

Omaveloxolone (2‐cyano‐3,12‐dioxooleana‐1,9‐dien‐28‐oic acid‐difluoro‐propyl‐amide [CDDO‐DFPA]; RTA 408), a semisynthetic oleanane triterpenoid, is in a class of compounds known to be very potent activators of nuclear factor, erythroid 2 like 2 (Nrf2).^[^ ^1^ ^]^ Nrf2 is the master transcriptional regulator of the antioxidative response and has a multitude of cytoprotective functions. For example, it is widely established that Nrf2 transcriptionally controls the expression of proteins involved in glutathione synthesis, detoxification of reactive oxygen species (ROS), inhibition of inflammation, and repair and removal of damaged proteins. More recent studies have shown that Nrf2 also regulates mitochondrial function and bioenergetics.^[^ ^2^ ^]^ For example, the detoxification of ROS extends beyond the cytoplasm into the mitochondria, where ROS homeostasis is critical to maintaining proper mitochondrial function and ATP production. Nrf2 can also be physically bound to mitochondria, which aids in its ability to rapidly respond to alterations in mitochondrial function.^[^ ^3^ ^]^ In addition to responding to ROS, Nrf2 can increase the efficient use of fatty acids and glucose by affecting fundamental pathways of metabolism.^[^ ^4^ ^]^ For example, Nrf2 enhances the expression of genes encoding enzymes involved in the pentose phosphate pathway, which uses glucose to produce the reduced form of nicotinamide adenine dinucleotide phosphate, an important reducing cosubstrate for many antioxidative reactions, including the reductive recycling of oxidized glutathione.^[^ ^5^ ^]^ Nrf2 also decreases the levels of acetyl‐CoA carboxylase, the rate‐limiting enzyme in fatty acid synthesis, which decreases the levels of malonyl‐CoA, and results in increased fatty acid oxidation by relieving the suppression of carnitine palmitoyltransferase 1 (Cpt1).^[^ ^6^ ^]^ Cpt1 catalyzes the rate‐limiting step in the beta‐oxidation of long‐chain fatty acids, and Nrf2 is essential for Cpt1 expression, particularly in settings of high‐fat diet (HFD) and oxidative stress.^[^ ^7^ ^]^

Paramount to the effects of Nrf2 on mitochondrial function, a pivotal clinical trial of omaveloxolone in patients with Friedreich's ataxia was recently completed.^[^ ^8^, ^9^ ^]^ Friedreich's ataxia is a disease caused by a deficiency in frataxin, a protein involved in the assembly of iron–sulfur clusters, which leads to increased free radicals, oxidative stress, and disruption of mitochondrial homeostasis primarily in the cerebellum, liver, and heart. Administration of omaveloxolone once daily for 48 weeks to patients with Friedreich's ataxia significantly increased the modified Friedreich's ataxia Rating Scale score when compared with a placebo control group.^[^ ^8^, ^9^ ^]^ Further mechanistic support of the effects of omaveloxolone in the setting of mitochondrial dysfunction comes from studies in Friedreich's ataxia mouse and patient cell models. Omaveloxolone improved mitochondrial function and ATP generation and protected against oxidative stress in neurons from KIKO and YG8R Friedreich's ataxia mice and also in fibroblasts from patients with Friedreich's ataxia.^[^ ^10^ ^]^ Consistent with the noteworthy improvements observed in Friedreich's ataxia patients and the known cytoprotective effects of Nrf2 in the central nervous system,^[^ ^11^ ^]^ omaveloxolone has also demonstrated efficacy in rodent models of status epilepticus^[^ ^12^ ^]^ and multiple sclerosis.^[^ ^13^ ^]^ Furthermore, in line with the broad applicability of its effects on oxidative stress, inflammation, and mitochondrial dysfunction, omaveloxolone has also demonstrated efficacy in animal models of diseases outside of the central nervous system. For example, omaveloxolone demonstrates significant efficacy in rodent models of kidney ischemia‐reperfusion injury, multiple sclerosis, diabetic wound healing, and radiation‐induced dermatitis.^[^ ^13^, ^14^, ^15^, ^16^ ^]^

Nonalcoholic fatty liver disease (NAFLD) is the most common liver disease, affecting 80–100 million people in the United States.^[^ ^17^ ^]^ Approximately, 25% of cases of NAFLD progress to nonalcoholic steatohepatitis (NASH), which is defined by steatosis, inflammation, cellular ballooning, and fibrosis.^[^ ^17^ ^]^ NASH has the potential to progress to cirrhosis and hepatocellular carcinoma and is associated with many other comorbidities, such as colon cancer and chronic kidney disease.^[^ ^18^ ^]^ Mitochondria control hepatic lipid metabolism and regulate oxidative stress in the liver, and, as such, NAFLD and NASH are associated with the disruption of mitochondrial homeostasis, often as a consequence of type 2 diabetes and/or obesity.^[^ ^19^ ^]^ Because mitochondrial dysfunction leads to increased production of ROS and inflammation and decreased fatty acid oxidation, the role of mitochondria appears to be central to the mechanism contributing to the pathogenesis of NAFLD and NASH.^[^ ^20^ ^]^

**Figure 1:**
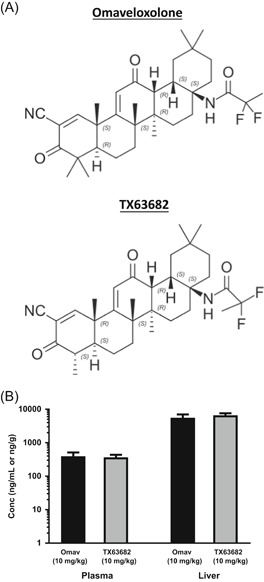
Chemical structures and plasma and liver concentrations of omaveloxolone and analog TX63682. A, Chemical structures of omaveloxolone and TX63682 are presented. B, Blood and liver were collected 4 hours following the final dose after 14 daily oral doses of omaveloxolone (n = 8) or TX63682 (n = 5 for plasma and n = 10 for liver) at a dose level of 10 mg/kg to male C57Bl/6 mice. Plasma and liver concentrations were determined using liquid chromatography‐tandem mass spectrometry methodology. Plasma concentrations are presented as mean ng/mL ± SD, and liver concentrations are presented as mean ng/g ± SD. Omav, omaveloxolone

Therefore, because Nrf2 is critical for mitochondrial homeostasis, lipid metabolism, and overall hepatoprotection,^[^ ^21^ ^]^ the effects of omaveloxolone and a closely related analog, TX63682, were investigated in the STAM mouse model of NASH.^[^ ^22^ ^]^ Diabetes and obesity are key features of the STAM model, which has been described to recapitulate the known progression of the human disease.^[^ ^23^ ^]^ Structures of omaveloxolone and TX63682 are presented in Figure 1A. In this model, NASH is induced by a single injection of the pancreatic β‐cell toxin streptozotocin (STZ) at 2 days after birth with HFD initiated at 4 weeks of age. This NASH model is characterized by insulin resistance, hyperglycemia, dyslipidemia, and accumulation of hepatic lipids, which facilitates oxidative stress, inflammation, and liver fibrosis.^[^ ^24^ ^]^

## 2 METHODS

### 2.1 Animals

For the plasma drug concentration and liver content study, C57Bl/6 mice were obtained from Charles River Laboratories (Wilmington, MA). The study was approved by the Institutional Animal Care and Use Committee at the laboratory where the in‐life study was conducted (Biomodels Inc, Watertown, MA). Mice were administered omaveloxolone or TX63682 at 10 mg/kg once daily for 14 days. Sesame oil was used as the vehicle with a dosing volume of 10 mL/kg. Mice were terminated 4 hours after the final dose, and plasma (supplemented with sodium sulfite, 0.25% wt/vol) and livers were collected for determination of drug concentrations.

The in‐life portion of the NASH study was conducted at Stelic Institute & Co, (Tokyo, Japan). Thirty C57Bl/6 mice (15‐day‐pregnant females) were obtained from Charles River Laboratories Japan (Kanagawa, Japan). All animals were housed and cared for in accordance with the Japanese Pharmacological Society Guidelines for Animal Use. NASH was induced in 68 male mice by a single subcutaneous injection of 20 μL STZ (Catalog #S0130, Lot #031M1287V; Sigma‐Aldrich) solution (10 mg/mL) 2 days after birth, followed by feeding with HFD (57 kcal% fat, Catalog #HFD32; CLEA Japan Inc, Japan) ad libitum from 4 to 9 weeks of age. The mice were randomized into a vehicle group (n = 6) and drug treatment groups (n = 8/group) at 6 weeks of age. Male littermates (n = 6), without STZ treatment and fed with normal diet ad libitum, were used for the normal group. Omaveloxolone (1, 3, or 10 mg/kg), TX63682 (1, 3, or 10 mg/kg), or sesame oil vehicle (10 mL/kg) were administered by oral gavage once daily from 6 to 9 weeks of age. Mice were terminated at 9 weeks of age, and whole blood, serum, liver, and pancreas were collected for the various biochemical and molecular biology assays listed in the following sections.

### 2.2 Quantification of drug concentrations in plasma and liver

Omaveloxolone and TX63682 were extracted from mouse plasma and, then, quantified using liquid chromatography‐tandem mass spectrometry (LC‐MS/MS) methods similar to those described previously.^[^ ^25^, ^26^, ^27^ ^]^ Plasma calibration curves were prepared using blank C57Bl/6 mouse plasma obtained from a commercial supplier (BioIVT, Westbury, NY). All plasma samples and controls were supplemented with 0.25% sodium sulfite to prevent oxidative degradation of omaveloxolone or TX63682. Omaveloxolone and TX63682 were extracted from mouse livers and, then, quantified using liquid chromatography methods similar to those previously described.^[^ ^25^ ^]^

### 2.3 Blood biochemistry

Nonfasting blood glucose levels were measured in whole blood samples using G‐Checker (Sanko Junyaku, Japan). Glycated hemoglobin A_1c_ (HbA_1c_) levels were quantified by a DCA Vantage Analyzer (Siemens Healthcare Diagnostics, IL) at Shin Nippon Biomedical Laboratories Ltd, (Kagoshima, Japan). For serum biochemistry, blood was collected in polypropylene tubes without anticoagulant and kept at room temperature for 30 minutes, followed by 4°C for 1 hour. The blood samples were then centrifuged at 1000 x *g* for 15 minutes at 4°C. The supernatant was collected and stored at −80°C until use. Serum levels of alanine transaminase (ALT), aspartate transaminase (AST), total bilirubin, and gamma‐glutamyl transpeptidase (GGT) were measured using a FUJI DRI‐CHEM7000 chemistry analyzer (Fujifilm, Japan). Serum total cholesterol, high‐density lipoprotein (HDL) cholesterol, low‐density lipoprotein (LDL) cholesterol, and triglyceride levels were analyzed by high‐performance liquid chromatography at Skylight Biotech (Akita, Japan) using proprietary validated methods. Serum insulin, leptin, and adiponectin concentrations were quantified by the Ultra Sensitive Mouse Insulin ELISA Kit (Catalog #200761, Lot #12MAUM1214; Morinaga Institute of Biological Science, Japan), Mouse Leptin ELISA Kit (Catalog #MS331, Lot #12MAML338; Morinaga Institute of Biological Science), and Mouse Adiponectin Quantikine ELISA Kit (Catalog #MRP300, Lot #510‐87851; R&D Systems).

### 2.4 Measurement of liver triglycerides

Liver total lipid extracts were obtained from right lobes by Folch's method.^[^ ^28^ ^]^ The liver lobes were homogenized in chloroform‐methanol (2:1, vol/vol) and incubated overnight at room temperature. After washing with chloroform‐methanol‐water (8:4:3, vol/vol/vol), the extracts were evaporated to dryness and dissolved in isopropanol. Liver triglyceride content was measured by the triglyceride E‐test (Catalog #432‐40201, Lot #TJ482; Wako Pure Chemical Industries, Japan). When the triglyceride content exceeded the detection limit, extracts were diluted twofold in isopropanol and were then reanalyzed.

### 2.5 Histopathology

For liver hematoxylin and eosin (H&E) staining, sections were cut from paraffin blocks of liver tissue prefixed in Bouin's solution and stained with Lillie‐Mayer's hematoxylin (Muto Pure Chemicals, Japan) and eosin solution (Wako Pure Chemical Industries). NAFLD activity scores were calculated according to the criteria of Kleiner.^[^ ^29^ ^]^ To visualize collagen deposition, Bouin's fixed liver sections were stained using picro‐sirius red solution (Waldeck GmbH & Co, KG, Germany). To visualize macro‐ and microvesicular fat, cryosections were cut from left lateral livers embedded in Tissue‐Tek O.C.T. compound (Sakura Finetek Japan, Japan) and stained with Oil Red O (Sigma‐Aldrich). For quantitative analysis of fibrosis or fat deposition areas, bright field images were captured using a digital camera (DFC280; Leica, Germany) around central veins at 200‐fold magnification, and the positive areas in 5 fields/section were quantified using ImageJ software (National Institutes of Health). For pancreas H&E staining, sections were cut from paraffin blocks of pancreatic head tissue prefixed in 4% paraformaldehyde solution and stained with Lillie–Mayer's hematoxylin and eosin solution. For immunohistochemistry of insulin, sections were cut from frozen pancreatic tail tissues embedded in Tissue‐Tek O.C.T. compound and fixed in acetone. Endogenous peroxidase activity was blocked using 0.03% H_2_O_2_ for 5 minutes, followed by incubation with Block Ace (Dainippon Sumitomo Pharma, Japan) for 10 minutes. The sections were incubated with a 100‐fold dilution of anti‐insulin antibody (Santa Cruz Biotechnology) overnight at 4°C. After an overnight incubation with secondary antibody (×25 diluted horseradish peroxidae‐goat anti‐rabbit antibody; Vector Laboratories), enzyme‐substrate reactions were performed using 3,3’‐diaminobenzidine/H_2_O_2_ solution (Nichirei, Japan). Nuclear counterstaining was performed using Lillie–Mayer's hematoxylin solution.

### 2.6 Messenger RNA quantification

Livers were homogenized at 16.67 mg tissue/mL homogenization buffer (Catalog #QS0106; Thermo Fisher Scientific) containing 0.5 mg/mL Proteinase K. After homogenization, samples were vortexed at high speed for 1 hour at 65°C. The samples were centrifuged at 16,000 ×*g* for 15 minutes at room temperature. The supernatant was then collected, diluted 30‐40‐fold in homogenization buffer to be within the linear dynamic range of the assay, and stored at −80°C until analysis. Messenger RNA (mRNA) was quantified using Quantigene Plex 2.0 technology according to manufacturer's protocol (Thermo Fisher Scientific) and as previously described.^[^ ^30^ ^]^ The design of probe targets for NAD(P)H:quinone oxidoreductase 1 (Nqo1), sulfiredoxin 1 (Srxn1), ferritin heavy chain 1 (Fth1), and Rpl13a mRNA is freely available at https://www.thermofisher.com/order/quantigene-plex/configuration. Raw values were normalized to Rpl13a and presented as fold mean normal control ± standard error of the mean (SEM).

### 2.7 Nqo1 enzyme activity

Livers were homogenized at 250 mg tissue/mL in ice‐cold phosphate‐buffered saline at pH 7.2 (Catalog #70013; Invitrogen), fortified with 2 mM ethylenediaminetetraacetic acid. Homogenates were then centrifuged at 10 000 x *g* for 10 minutes at 4°C. The supernatants were collected and stored at −80°C until analysis. Protein concentrations of tissue homogenates were determined using the Bicinchoninic Acid Protein Assay Kit from Pierce Biotechnology (Catalog #23225; Rockford, IL). Liver cytosolic Nqo1 enzyme activity was determined by quantifying the reduction of 2,6‐dichlorophenol‐indophenol in the presence and absence of dicumarol, as previously described^[^ ^31^ ^]^ and modified.^[^ ^32^ ^]^ Data were normalized to protein concentration and presented as fold mean normal control.

### 2.8 Statistics

Statistical analyses were performed using a one‐way analysis of variance followed by Dunnett's test for parametric data or Dunn's test for nonparametric data, where appropriate, using the Prism Software v7 (GraphPad Software). *P* value less than .05 was considered statistically significant. Results are expressed as mean ± SEM.

## 3 RESULTS

### 3.1 Omaveloxolone and TX63682 are orally bioavailable and similarly distribute to liver

After oral dosing of omaveloxolone or TX63682 to C57Bl/6 mice once daily for 14 days, plasma and liver samples were collected 4 hours after the final dose, and drug concentrations were determined by LC/MS/MS methods (Figure 1B). Overall, pharmacologically relevant concentrations were readily achieved, and exposures were similar between compounds.

### 3.2 Omaveloxolone and TX63682 do not affect body or liver weights in the STAM mouse model

Mean body weights for all groups of STAM mice were significantly lower than those of normal mice at the start and completion of the study (Table 1). There were no significant differences in mean body weight between the vehicle‐treated STAM mice and any of the drug‐treated STAM mice during the dosing period. Liver weights were also significantly higher in the vehicle‐treated STAM mice compared with the normal nondiseased mice (Table 1). Treatment with omaveloxolone or TX63682 also did not affect liver weight. Likewise, liver‐to‐body weight ratios were significantly higher in the vehicle‐treated STAM mice and were not affected by treatment with omaveloxolone or TX63682 (Table 1). The decrease in body weight and increase in liver weight between normal mice and vehicle‐treated STAM mice are consistent with previous findings.^[^ ^24^ ^]^

**Table 1:** Body weights, liver weights, liver‐to‐body weight ratios, and serum insulin

Dose group	Initial body weight, g	Final body weight, g	Liver weight, g	Liver:body weight (%)	Serum insulin, ng/mL
Normal	21.9 ± 0.5	23.7 ± 0.7**	1.042 ± 0.046**	4.4 ± 0.1**	0.62 ± 0.08**
Vehicle control	18.3 ± 1.3	19.3 ± 1.3	1.362 ± 0.097	7.1 ± 0.5	0.25 ± 0.12
Omaveloxolone, 1 mg/kg	18.4 ± 0.7	19.6 ± 0.8	1.381 ± 0.091	7.0 ± 0.5	0.31 ± 0.17
Omaveloxolone, 3 mg/kg	18.5 ± 0.7	19.4 ± 1.0	1.388 ± 0.153	7.2 ± 0.7	0.25 ± 0.18
Omaveloxolone, 10 mg/kg	18.3 ± 0.7	19.5 ± 1.2	1.379 ± 0.028	7.0 ± 1.1	0.34 ± 0.11
TX63682, 1 mg/kg	18.4 ± 0.6	19.9 ± 0.9	1.419 ± 0.199	7.1 ± 0.8	0.31 ± 0.21
TX63682, 3 mg/kg	18.3 ± 1.0	19.3 ± 1.4	1.419 ± 0.200	7.4 ± 0.7	0.23 ± 0.09
TX63682, 10 mg/kg	18.6 ± 1.2	19.2 ± 1.1	1.390 ± 0.140	7.3 ± 0.7	0.26 ± 0.13

*Note*: Data presented as (mean ± SD).

** *P * < .01 vs all STAM mice (vehicle control and all treated groups).

### 3.3 Omaveloxolone and TX63682 improve liver architecture and decrease collagen and fat deposits

**Figure 2:**
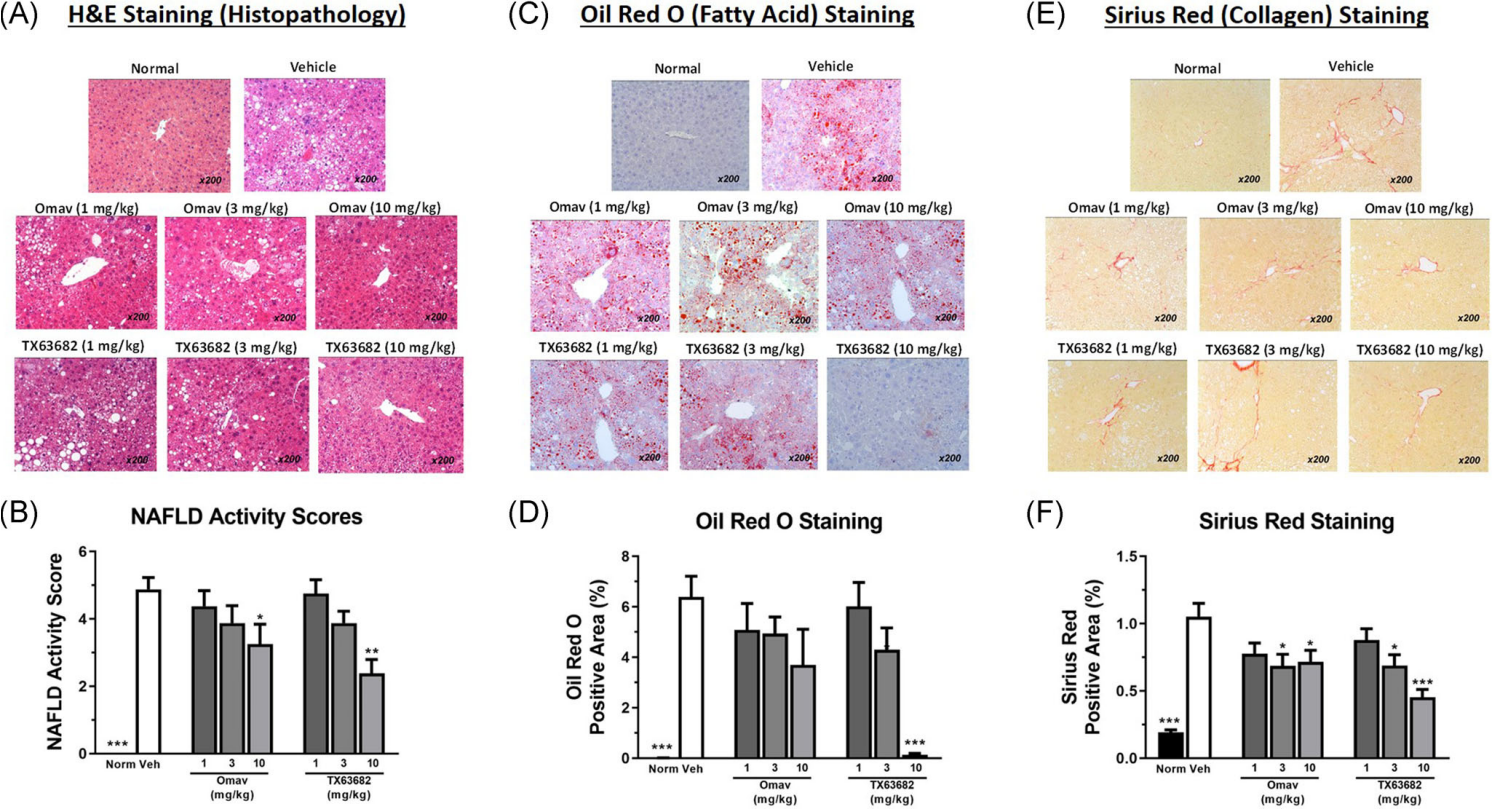
Omaveloxolone and analog TX63682 protect liver in the model of NASH. Representative photomicrographs are presented for histopathology (A), Oil Red O staining of fatty acids (C), and Sirius Red staining of collagen (E). NAFLD activity scores (B) and percent positive area for Oil Red O (D) and Sirius Red (F) are also presented. Data are shown as fold change from normal control ± SEM. Asterisks indicate a statistically significant difference from the vehicle control group (*P < .05, **P < .01, ***P < .001). H&E, hematoxylin and eosin; NAFLD, nonalcoholic fatty liver disease; NASH, nonalcoholic steatohepatitis; Omav, omaveloxolone

As expected in the STAM model of NASH,^[^ ^22^, ^24^ ^]^ livers from the vehicle group exhibited severe microvesicular and macrovesicular fat deposition, hepatocellular ballooning, and inflammatory cell infiltration, which were reflected by a marked increase in NAFLD activity scores compared to normal mice (Figure 2A,B). Treatment with either omaveloxolone or TX63682 ameliorated these histopathological features (Figure 2A) and resulted in dose‐dependent decreases in NAFLD activity scores with statistical significance achieved at 10 mg/kg for both compounds (Figure 2B).

Compared with livers from normal nondiseased mice, livers from vehicle‐treated STAM mice demonstrated significant increases in lipid accumulation in hepatocytes, as assessed by Oil Red O staining (Figure 2C,D). Omaveloxolone demonstrated a trend with apparent dose‐dependent decreases in lipid accumulation, though the difference in the mean percent positive area for Oil Red O staining was not statistically significant at any dose (Figure 2C,D). TX63682 also demonstrated decreases in lipid accumulation with a statistically significant difference observed at 10 mg/kg. In fact, the amount of lipid staining at 10 mg/kg TX63682 was similar to that of normal nondiseased mice. Consistent with other parameters of liver injury, the vehicle‐treated STAM mice also showed increased collagen deposition in the pericentral region of the liver lobule (Figure 2E,F). Both omaveloxolone and TX63682 decreased collagen deposition, with statistical significance observed at 3 and 10 mg/kg (Figure 2E,F).

### 3.4 Omaveloxolone and TX63682 induce Nrf2 target genes in liver

**Figure 3:**
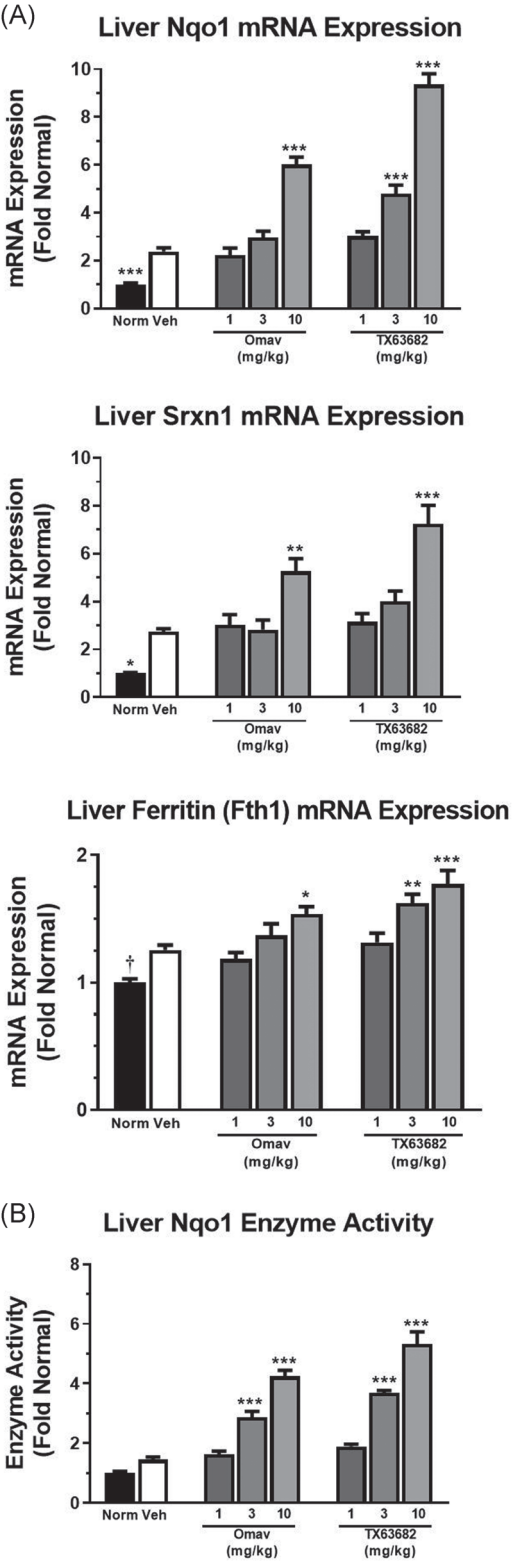
Omaveloxolone and analog TX63682 induce liver Nrf2 targets. A, The mRNA expression of Nqo1, Srxn1, and Fth1 was determined using Quantigene Plex 2.0 technology. Data are normalized to Rpl13a and are presented as fold change from normal control ± SEM. B, Liver Nqo1 enzyme activity was also evaluated by quantifying the reduction of DCPIP in the presence and absence of dicumarol. Enzyme activity data are presented as fold change from normal control ± SEM. Asterisks indicate a statistically significant difference from the vehicle control group (*P < .05, **P < .01, ***P < .001). DCPIP, 2,6‐dichlorophenol‐indophenol; mRNA, messenger RNA; Omav, omaveloxolone

Expression of the Nrf2 target genes Nqo1, Srxn1, and Fth1 was quantified in liver (Figure 3A). Nqo1 enzyme activity, another functional measurement of Nrf2 activity, was also evaluated in livers (Figure 3B). Vehicle‐treated STAM mice tended to have modest increases in Nrf2 target gene expression compared to normal control mice, likely as a consequence of the oxidative stress present in the disease model but clearly not sufficient enough to provide adequate hepatoprotection. Both omaveloxolone and TX63682 demonstrated dose‐dependent increases in Nrf2 target gene expression, with statistical significance observed at 3 mg/kg for some targets and at 10 mg/kg for all targets. Overall, these Nrf2 target gene data confirm engagement of Nrf2 by omaveloxolone and TX63682 in liver.

### 3.5 Omaveloxolone and TX63682 improve glucose control and alter lipid profiles

**Figure 4:**
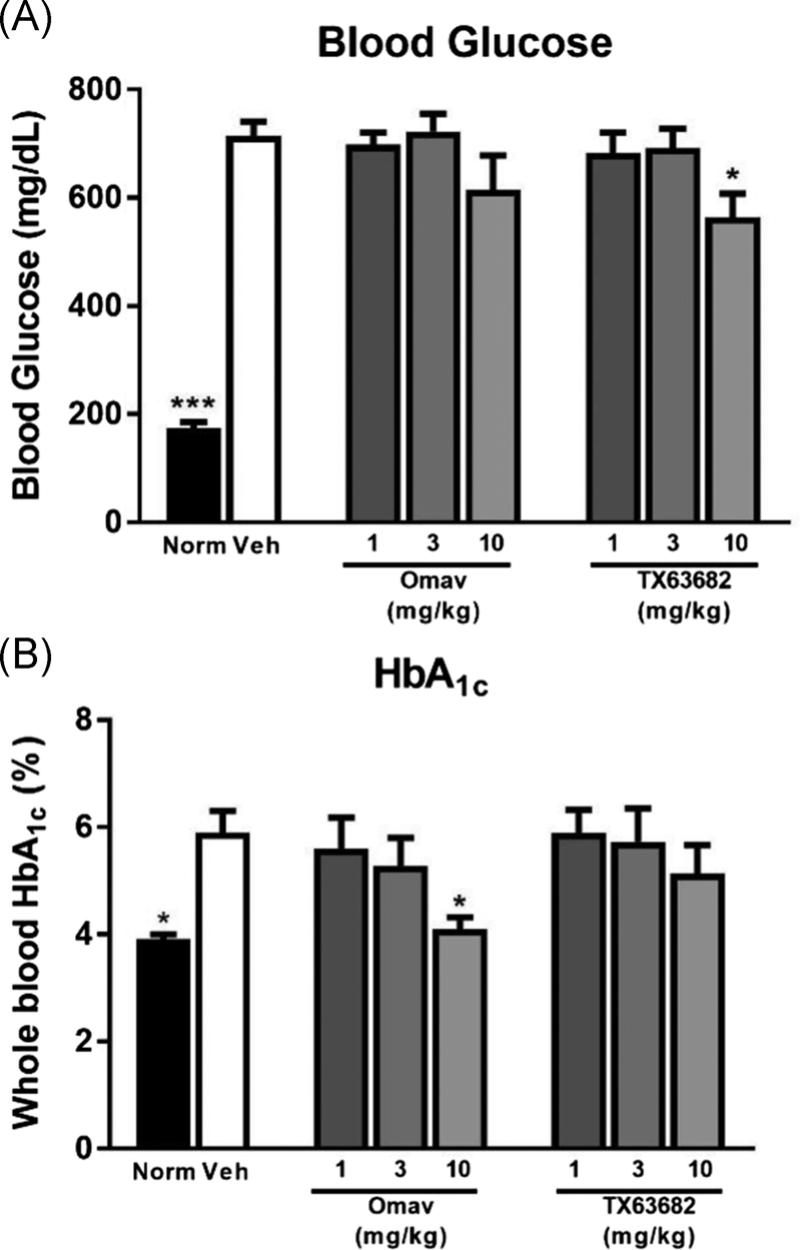
Effect of omaveloxolone and analog TX63682 on blood glucose and HbA_1c_. A, Blood glucose was determined using a G‐Checker (Sanko Junyaku, Japan). B, HbA_1c_ levels were quantified by DCA Vantage Analyzer, a point‐of‐care immunoassay analyzer. Data are presented as mean ± SEM. Asterisks indicate a statistically significant difference from the vehicle control group (*P < .05). HbA_1c_, hemoglobin A_1c_; Omav, omaveloxolone

Nonfasting blood glucose and serum glycated HbA_1c_ levels were significantly elevated in vehicle‐treated STAM mice compared to normal nondiseased mice (Figure 4A,B). Treatment with omaveloxolone or TX63682 at 10 mg/kg tended to decrease, or significantly decreased, blood glucose levels. Consistent with the decreases in fasting blood glucose, serum HbA_1c_ was also lower with both omaveloxolone and TX63682 at 10 mg/kg, with statistical significance observed with omaveloxolone.

**Figure 5:**
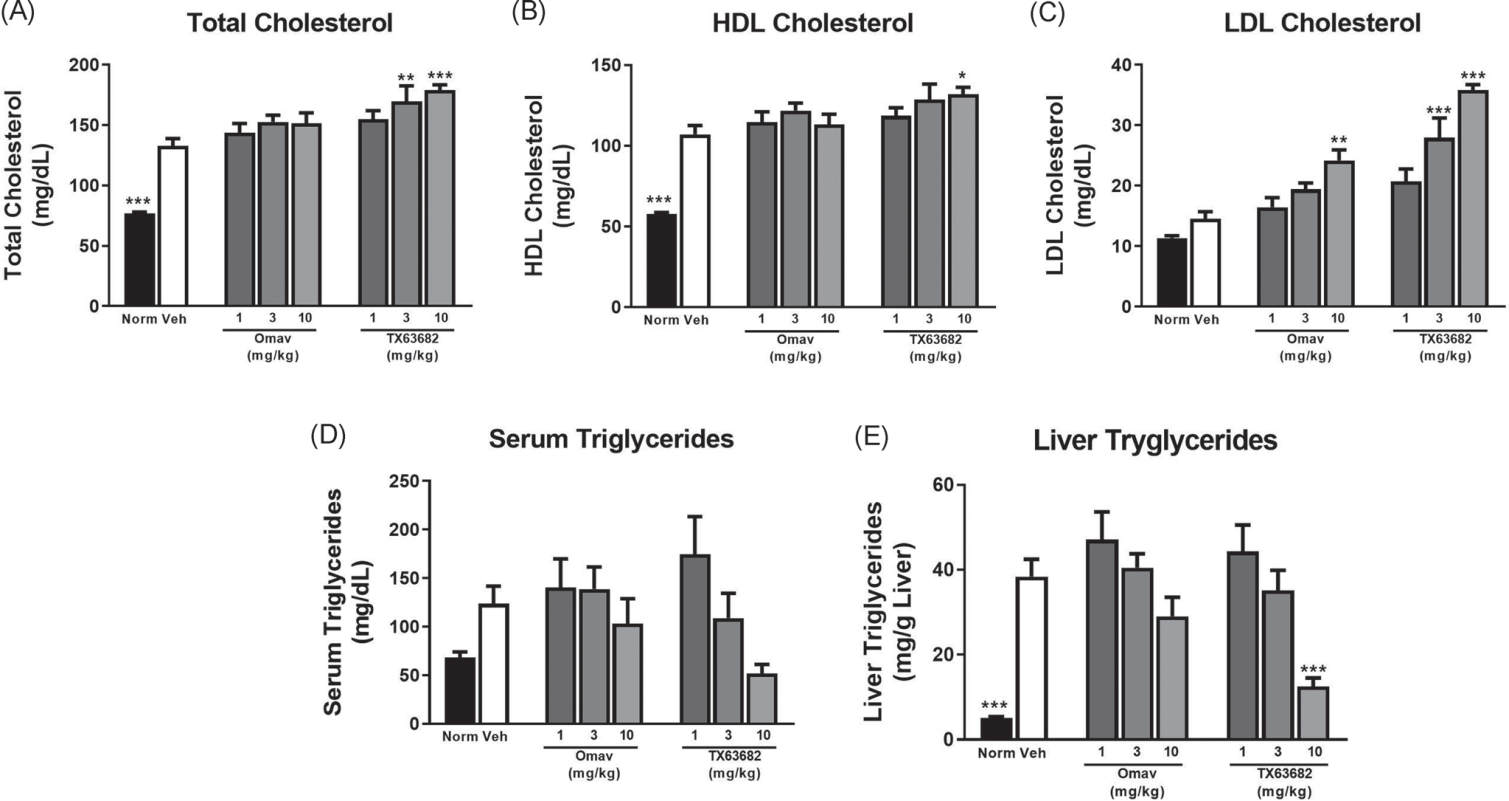
Effect of omaveloxolone and analog TX63682 on lipids. Serum total cholesterol (A), HDL cholesterol (B), LDL cholesterol (C), and triglycerides (D) were analyzed by HPLC. E, Liver triglycerides were quantified with the commercially available Triglyceride E‐test Kit (Wako Chemicals). Data are presented as mean ± SEM. Asterisks indicate a statistically significant difference from the vehicle control group (*P < .05, **P < .01, ***P < .001). HDL, high‐density lipoprotein; HPLC, high‐performance liquid chromatography; LDL, low‐density lipoprotein; Omav, omaveloxolone

Serum total cholesterol levels, HDL cholesterol, LDL cholesterol, serum triglycerides, and liver triglycerides were all higher in vehicle‐treated STAM mice compared to normal nondiseased mice (Figure 5A‐E). Omaveloxolone and TX63682 mildly and dose‐dependently increased serum total cholesterol with statistically significant increases observed at 3 and 10 mg/kg TX63682. Omaveloxolone and TX63682 only modestly increased HDL cholesterol (Figure 5B); however, both compounds significantly and dose‐dependently increased LDL cholesterol (Figure 5C). Reductions in both serum and liver triglycerides were observed for both omaveloxolone and TX63682 with a statistically significant decrease in liver triglycerides observed by TX63682 at 10 mg/kg (Figure 5D,E).

**Figure 6:**
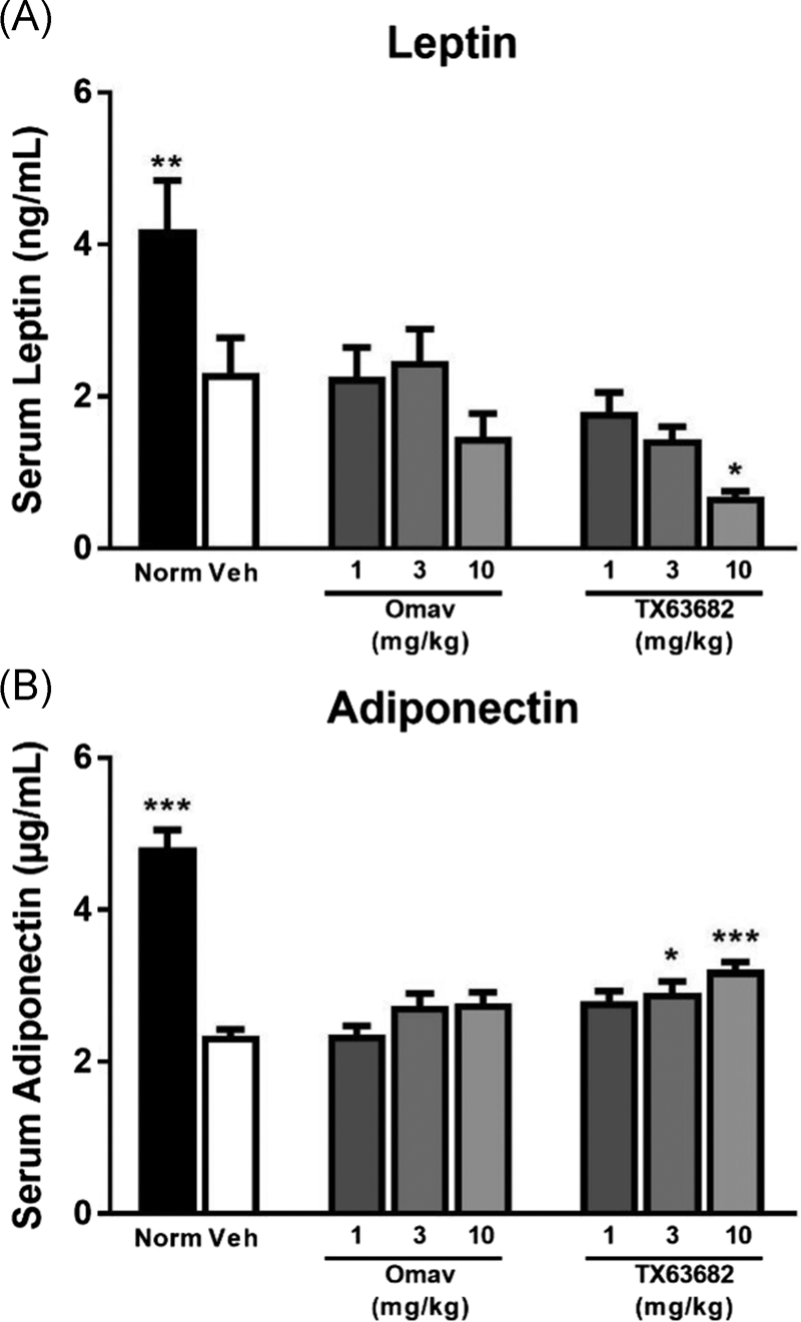
Effect of omaveloxolone and analog TX63682 on leptin and adiponectin. Serum leptin (A) and adiponectin (B) concentrations were quantified by ELISA. Data are presented as mean ± SEM. Asterisks indicate a statistically significant difference from the vehicle control group (*P < .05, **P < .01, ***P < .001). ELISA, enzyme‐linked immunosorbent assay; Omav, omaveloxolone

Both leptin and adiponectin were significantly decreased in vehicle‐treated STAM mice compared to normal nondiseased mice (Figure 6A,B). Both omaveloxolone and TX63682 at 10 mg/kg decreased leptin further with statistical significance obtained by TX63682. In contrast, both omaveloxolone and TX63682 slightly increased adiponectin compared to vehicle with statistical significance obtained by TX63682 at 3 and 10 mg/kg.

Pancreatic tissue from the vehicle‐treated STAM mice showed islet atrophy, a reduction in the number and sizes of islets, and fewer insulin‐positive β cells compared to pancreatic tissue from normal nondiseased mice (data not shown). However, no differences in pancreatic insulin staining, or pancreas histology, were observed between STAM mice treated with vehicle and STAM mice treated with any dose of omaveloxolone or TX63682 (data not shown). Serum insulin was also significantly lower in the vehicle‐treated STAM group compared to the normal group; however, no differences were observed between STAM mice treated with vehicle and STAM mice treated with any dose of omaveloxolone or TX63682 (Table 1). These data indicate that the effects of omaveloxolone and TX63682 on glucose and lipid levels were not related to islet status.

### 3.6 Omaveloxolone and TX63682 increase serum transaminases

**Figure 7:**
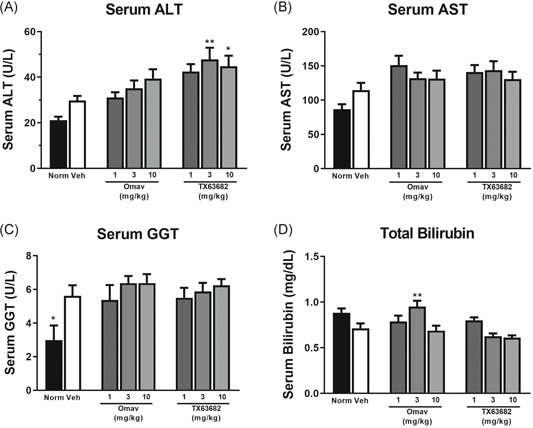
Effect of omaveloxolone and analog TX63682 on serum transaminases. Serum ALT (A), AST (B), GGT (C), and total bilirubin (D) were quantified using a FUJI DRI‐CHEM7000 chemistry analyzer. Data are presented as mean ± SEM. Asterisks indicate a statistically significant difference from the vehicle control group (*P < .05, **P < .01). ALT, alanine transaminase; AST, aspartate transaminase; GGT, gamma‐glutamyl transpeptidase; Omav, omaveloxolone

Compared to normal nondiseased mice, vehicle‐treated STAM mice had mild increases in both serum ALT and AST (Figure 7A,B). Treatment with omaveloxolone or TX63682 resulted in mild and dose‐dependent increases in ALT with statistical significance obtained by TX63682 (Figure 7A). Mild increases in serum AST were also observed with both omaveloxolone and TX63682 treatment (Figure 7B). No meaningful differences among any of the NASH treatment groups were observed for serum GGT, though there were mild but significant elevations between the vehicle‐treated NASH and normal mice (Figure 7C). There was a minimal yet statistically significant increase in total bilirubin at 3 mg/kg omaveloxolone but no changes at 10 mg/kg, and mean total bilirubin trended lower in the 3 and 10 mg/kg dose groups for TX63682 (Figure 7D). Based on the evidence, the minimal change observed at 3 mg/kg omaveloxolone was considered a statistical anomaly and not biologically meaningful. Overall, the mild increases in serum ALT and AST occurred in the absence of meaningful changes in bilirubin and were concurrent with marked improvements in hepatic histology, lipid accumulation, and collagen deposition (Figure 2).

## 4 DISCUSSION

Omaveloxolone and its analog TX63682 demonstrated significant hepatoprotection in the STAM mouse model of NASH, as evidenced by improvements in multiple parameters of hepatic physiology. At doses that significantly and dose‐dependently increased Nrf2 target gene expression in the liver, both compounds decreased hepatocellular ballooning and inflammation, as well as lipid accumulation and collagen deposition. Enhanced blood glucose control and reduced serum and liver triglycerides were also observed. Consistent with its higher potency in vitro (not shown for TX63682, see previous data for omaveloxolone^[^ ^33^ ^]^), TX63682 performed slightly better than omaveloxolone in this model, despite achieving similar plasma and liver exposures after oral dosing. The higher activity of TX63682 was associated with higher Nrf2 target gene induction in liver.

Interestingly, despite the improvements in hepatic lipid profiles with omaveloxolone and TX63682, mild, and in some cases significant, increases in serum total, HDL, and LDL cholesterol were observed. Another analog of omaveloxolone, CDDO‐Im, also mildly increased serum cholesterol in an HFD mouse model of obesity, while attenuating weight gain and hepatic fat accumulation and increasing energy expenditure and oxygen consumption.^[^ ^34^ ^]^ Strikingly, CDDO‐Im did not affect serum cholesterol or produce the profound improvements on obesity in Nrf2‐null mice. Consistent with these observations, untreated Nrf2‐null mice have lower serum cholesterol levels than wild‐type mice when fed an HFD.^[^ ^35^ ^]^ Overall, these data suggest that Nrf2 plays an important role in the regulation of cholesterol homeostasis. The mechanism underlying this phenomenon is currently unknown, but we speculate that it may be related to the known improvements in lipid handling, fat mobilization, fatty acid oxidation, and mitochondrial function that have been shown to occur with omaveloxolone treatment and Nrf2 activation.^[^ ^6^ ^]^ Additionally, the omaveloxolone analog CDDO‐dhTFEA increases glutathione‐dependent bile flow and the biliary excretion of cholesterol,^[^ ^36^ ^]^ and Nrf2 regulates the expression of the cholesterol efflux transporters ATP‐binding cassette sub‐family G member 5 (Abcg5) and Abcg8.^[^ ^37^ ^]^ These phenomena may also contribute to compensatory mechanisms that result in mild increases in serum cholesterol.

Mild increases in serum transaminases were observed in conjunction with clear improvements in hepatic histology and without major changes in serum bilirubin. These observations are consistent with the clinical profile of omaveloxolone and bardoxolone methyl, where reversible increases in serum transaminases are observed without increases in other markers of hepatic injury (Lewis, 2020, in preparation). Such increases in serum transaminases without apparent liver injury are postulated to be related to the pharmacology of these Nrf2 activators. Serum transaminase levels and hepatic transaminase gene expression have been observed to correlate with Nrf2 status in Nrf2‐null, wild‐type, and Keap1‐knockdown mice (Lewis, 2020, in preparation). Additionally, bardoxolone methyl increases mRNA levels of both ALT and AST isoforms in a variety of cell lines derived from hepatic and extrahepatic tissues (Lewis, 2020, in preparation). The physiological roles of transaminases have been reviewed.^[^ ^38^ ^]^ To briefly summarize, ALT and AST catalyze the reversible transfer of amino groups between alanine or aspartate, respectively, and α‐ketoglutarate to form glutamate and pyruvate (ALT) or oxaloacetate (AST). Because the substrates and products of the ALT and AST enzymatic reactions are critical for a variety of cellular functions, both ALT and AST contribute to other cellular processes outside of basic amino acid metabolism. ALT plays an important role in the glucose‐alanine cycle, which, in hepatocytes, facilitates pyruvate production that is then used to synthesize glucose. ALT also contributes glutamate to make glutathione,^[^ ^39^ ^]^ an important cellular antioxidant that is regulated by Nrf2. AST, on the contrary, is an important enzyme in the malate‐aspartate shuttle and the generation of NADH, a necessary cosubstrate for ATP generation by mitochondria.^[^ ^38^ ^]^

Induction of NASH in vehicle‐treated STAM mice led to significant decreases in both serum leptin and adiponectin compared to normal nondiseased mice. Treatment with omaveloxolone or TX63682 further decreased leptin values, while increasing adiponectin values back toward normal levels. Leptin, an adipokine, is known to contribute to hepatic disrepair and fibrogenesis often observed in response to liver injury.^[^ ^40^ ^]^ Leptin has also been shown to suppress Nrf2 activity, and the decrease in leptin observed with omaveloxolone or TX63682 treatment in this study is considered to be hepatoprotective and potentially related, at least in part, to the reduction in collagen deposition that was observed.^[^ ^41^ ^]^ Adiponectin is a potent anti‐inflammatory adipocytokine that has suppressive effects on tumor necrosis factor‐α‐mediated nuclear factor κB activity and has been shown to prevent liver fibrosis.^[^ ^42^ ^]^ As such, lower adiponectin levels are also considered to be an independent risk factor for NASH.^[^ ^43^ ^]^

The hepatoprotection observed with omaveloxolone and TX63682 in the STAM model of NASH presented herein is consistent with data from other models of liver injury that evaluated various analogs of omaveloxolone. For example, an analog suppressed hepatic steatosis and liver fibrosis in mice fed a high‐fat/high‐fructose diet.^[^ ^44^ ^]^ However, the STAM model of NASH is considered to be more clinically relevant because the progression of disease closely mirrors the known progression in humans but within a much shorter time frame.^[^ ^23^ ^]^ Significant hepatoprotection with omaveloxolone analogs has also been demonstrated in acetaminophen‐induced hepatotoxicity,^[^ ^45^ ^]^ chronic liver injury from carbon tetrachloride,^[^ ^46^ ^]^ and ischemia‐reperfusion.^[^ ^47^ ^]^ Thus, the overall hepatoprotective pharmacology of omaveloxolone and its analogs are consistent with the profound hepatoprotective properties of Nrf2, which has been studied and reviewed extensively.^[^ ^21^, ^48^, ^49^, ^50^, ^51^, ^52^ ^]^

In conclusion, omaveloxolone and TX63682 demonstrated significant hepatoprotection in a rodent model of NASH. Such hepatoprotection was observed in conjunction with improvements in glucose control and lipid handling, which is consistent with the overall phenotype of improved mitochondrial function that is associated with Nrf2 activation. Overall, these data suggest that omaveloxolone and its analogs may be useful for the treatment of liver diseases.

## References

[1] K. T. Liby , M. B. Sporn , Pharmacol. Rev. 2012, 64, 972. 22966038 10.1124/pr.111.004846 PMC3462991

[2] A. T. Dinkova‐Kostova , A. Y. Abramov , Free Radic. Biol. Med. 2015, 88, 179. 25975984 10.1016/j.freeradbiomed.2015.04.036 PMC4726722

[3] S. C. Lo , M. Hannink , Exp. Cell Res. 2008, 314, 1789. 18387606 10.1016/j.yexcr.2008.02.014 PMC2409987

[4] M. H. Ludtmann , P. R. Angelova , Y. Zhang , A. Y. Abramov , A. T. Dinkova‐Kostova , Biochem. J. 2014, 457, 415. 24206218 10.1042/BJ20130863 PMC4208297

[5] K. C. Wu , J. Y. Cui , C. D. Klaassen , Toxicol. Sci 2011, 123, 590. 21775727 10.1093/toxsci/kfr183 PMC3179677

[6] K. M. Holmstrom , R. V. Kostov , A. T. Dinkova‐Kostova , Curr. Opin. Toxicol. 2016, 1, 80. 28066829 10.1016/j.cotox.2016.10.002 PMC5193490

[7] Y. Tanaka , T. Ikeda , K. Yamamoto , H. Ogawa , T. Kamisako , J. Gastroenterol. Hepatol. 2012, 27, 1711. 22591204 10.1111/j.1440-1746.2012.07180.x

[8] S. D. Ghanekar , W. W. Miller , C. J. Meyer , K. J. Fenelon , A. Lacdao , T. A. Zesiewicz , Degener. Neurol. Neuromuscul. Dis. 2019, 9, 103. 31686946 10.2147/DNND.S180027 PMC6800542

[9] Reata Pharmaceuticals . Reata Announces Positive Topline Results from the MOXIe Registrational Trial of Omaveloxolone in Patients with Friedreich's Ataxia . Vol. 2019, 2019. https://www.reatapharma.com/press-releases/reata-announces-positive-topline-results-from-the-moxie-registrational-trial-of-omaveloxolone-in-patients-with-friedreichs-ataxia/. (accessed: 2 May 2020).

[10] R. Abeti , A. Baccaro , N. Esteras , P. Giunti , Front. Cell. Neurosci. 2018, 12, 188. 30065630 10.3389/fncel.2018.00188 PMC6056642

[11] A. D. Kraft , J. M. Lee , D. A. Johnson , Y. W. Kan , J. A. Johnson , J. Neurochem. 2006, 98, 1852. 16945104 10.1111/j.1471-4159.2006.04019.x

[12] T. Shekh‐Ahmad , R. Eckel , S. Dayalan Naidu , M. Higgins , M. Yamamoto , A. T. Dinkova‐Kostova , S. Kovac , A. Y. Abramov , M. C. Walker , Brain 2018, 141, 1390. 29538645 10.1093/brain/awy071

[13] H. J. Wei , T. K. Pareek , Q. Liu , J. J. Letterio , Sci. Rep. 2017, 7, 9886. 28851867 10.1038/s41598-017-06907-4 PMC5575165

[14] P. Han , Z. Qin , J. Tang , Z. Xu , R. Li , X. Jiang , C. Yang , Q. Xing , X. Qi , M. Tang , J. Zhang , B. Shen , W. Wang , C. Qin , W. Zhang , Oxid. Med. Cell Longev. 2017, 2017, 7612182. 29435098 10.1155/2017/7612182 PMC5757134

[15] P. S. Rabbani , T. Ellison , B. Waqas , D. Sultan , S. Abdou , J. A. David , J. M. Cohen , A. Gomez‐Viso , G. Lam , C. Kim , J. Thomson , D. J. Ceradini , Diabetes Res. Clin. Pract. 2018, 139, 11. 29476889 10.1016/j.diabres.2018.02.021

[16] S. A. Reisman , C. Y. Lee , C. J. Meyer , J. W. Proksch , S. T. Sonis , K. W. Ward , Radiat. Res. 2014, 181, 512. 24720753 10.1667/RR13578.1

[17] B. J. Perumpail , M. A. Khan , E. R. Yoo , G. Cholankeril , D. Kim , A. Ahmed , World J. Gastroenterol. 2017, 23, 8263. 29307986 10.3748/wjg.v23.i47.8263 PMC5743497

[18] J. Tong , J. J. Guo , Eur. Rev. Med. Pharmacol. Sci. 2019, 23, 8515. 31646583 10.26355/eurrev_201910_19165

[19] A. Mansouri , C. H. Gattolliat , T. Asselah , Gastroenterology 2018, 155, 629. 30012333 10.1053/j.gastro.2018.06.083

[20] J. Lee , J. S. Park , Y. S. Roh , Arch. Pharm. Res. 2019, 42, 935. 31571145 10.1007/s12272-019-01178-1

[21] C. D. Klaassen , S. A. Reisman , Toxicol. Appl. Pharmacol. 2010, 244, 57. 20122946 10.1016/j.taap.2010.01.013 PMC2860427

[22] M. Fujii , Y. Shibazaki , K. Wakamatsu , Y. Honda , Y. Kawauchi , K. Suzuki , S. Arumugam , K. Watanabe , T. Ichida , H. Asakura , H. Yoneyama , Med. Mol. Morphol. 2013, 46, 141. 23430399 10.1007/s00795-013-0016-1

[23] K. Takakura , T. Oikawa , Y. Tomita , Y. Mizuno , M. Nakano , C. Saeki , Y. Torisu , M. Saruta , World J. Gastroenterol. 2018, 24, 1989. 29760542 10.3748/wjg.v24.i18.1989 PMC5949712

[24] T. Saito , M. Muramatsu , Y. Ishii , Y. Saigo , T. Konuma , Y. Toriniwa , K. Miyajima , T. Ohta , Physiol. Res. 2017, 66, 791. 28730823 10.33549/physiolres.933592

[25] S. A. Reisman , S. S. Gahir , C. I. Lee , J. W. Proksch , M. Sakamoto , K. W. Ward , Drug Des. Devel. Ther. 2019, 13, 1259. 10.2147/DDDT.S193889 PMC6475100 31118567

[26] S. A. Reisman , A. R. Goldsberry , C. Y. Lee , M. L. O'Grady , J. W. Proksch , K. W. Ward , C. J. Meyer , BMC Dermatol. 2015, 15, 10. 26170027 10.1186/s12895-015-0029-7 PMC4501113

[27] S. A. Reisman , C. Y. Lee , C. J. Meyer , J. W. Proksch , K. W. Ward , Arch. Dermatol. Res. 2014, 306, 447. 24362512 10.1007/s00403-013-1433-7

[28] J. Folch , M. Lees , G. H. Sloane Stanley , J. Biol. Chem. 1957, 226, 497. 13428781

[29] D. E. Kleiner , E. M. Brunt , M. Van Natta , C. Behling , M. J. Contos , O. W. Cummings , L. D. Ferrell , Y. C. Liu , M. S. Torbenson , A. Unalp‐Arida , M. Yeh , A. J. McCullough , A. J. Sanyal , Hepatology 2005, 41, 1313. 15915461 10.1002/hep.20701

[30] S. A. Reisman , R. L. Yeager , M. Yamamoto , C. D. Klaassen , Toxicol. Sci. 2009, 108, 35. 19129213 10.1093/toxsci/kfn267 PMC2644398

[31] A. M. Benson , M. J. Hunkeler , P. Talalay , Proc. Natl. Acad. Sci. U. S. A. 1980, 77, 5216. 6933553 10.1073/pnas.77.9.5216 PMC350028

[32] L. M. Aleksunes , M. Goedken , J. E. Manautou , World J. Gastroenterol. 2006, 12, 1937. 16610002 10.3748/wjg.v12.i12.1937 PMC4087521

[33] B. L. Probst , I. Trevino , L. McCauley , R. Bumeister , I. Dulubova , W. C. Wigley , D. A. Ferguson , PLoS One 2015, 10, e0122942. 25897966 10.1371/journal.pone.0122942 PMC4405374

[34] S. Shin , J. Wakabayashi , M. S. Yates , N. Wakabayashi , P. M. Dolan , S. Aja , K. T. Liby , M. B. Sporn , M. Yamamoto , T. W. Kensler , Eur. J. Pharmacol. 2009, 620, 138. 19698707 10.1016/j.ejphar.2009.08.022 PMC2752754

[35] Y. Tanaka , L. M. Aleksunes , R. L. Yeager , M. A. Gyamfi , N. Esterly , G. L. Guo , C. D. Klaassen , J. Pharmacol. Exp. Ther. 2008, 325, 655. 18281592 10.1124/jpet.107.135822

[36] S. A. Reisman , K. W. Ward , C. D. Klaassen , C. J. Meyer , Xenobiotica 2013, 43, 571. 23244591 10.3109/00498254.2012.750022

[37] T. Kamisako , Y. Tanaka , Y. Kishino , T. Ikeda , K. Yamamoto , S. Masuda , H. Ogawa , J. Clin. Biochem. Nutr. 2014, 54, 90. 24688217 10.3164/jcbn.13-92 PMC3947974

[38] M. R. McGill , EXCLI J. 2016, 15, 817. 28337112 10.17179/excli2016-800 PMC5318690

[39] J. J. Ellinger , I. A. Lewis , J. L. Markley , J. Biomol. NMR 2011, 49, 221. 21380856 10.1007/s10858-011-9481-9 PMC3081430

[40] I. A. Leclercq , G. C. Farrell , R. Schriemer , G. R. Robertson , J. Hepatol. 2002, 37, 206. 12127425 10.1016/s0168-8278(02)00102-2

[41] Y. Tang , A. Chen , Lab. Invest. 2014, 94, 503. 24614199 10.1038/labinvest.2014.42 PMC4006284

[42] Y. Matsuzawa , Curr. Pharm. Des. 2010, 16, 1896. 20370675 10.2174/138161210791208893

[43] Y. Kamada , T. Takehara , N. Hayashi , J. Gastroenterol. 2008, 43, 811. 19012034 10.1007/s00535-008-2213-6

[44] R. S. Sharma , D. J. Harrison , D. Kisielewski , D. M. Cassidy , A. D. McNeilly , J. R. Gallagher , S. V. Walsh , T. Honda , R. J. McCrimmon , A. T. Dinkova‐Kostova , M. L. J. Ashford , J. F. Dillon , J. D. Hayes , Cell. Mol. Gastroenterol. Hepatol. 2018, 5, 367. 29552625 10.1016/j.jcmgh.2017.11.016 PMC5852394

[45] S. A. Reisman , D. B. Buckley , Y. Tanaka , C. D. Klaassen , Toxicol. Appl. Pharmacol. 2009, 236, 109. 19371629 10.1016/j.taap.2008.12.024 PMC2680225

[46] Y. Getachew , F. A. Cusimano , P. Gopal , S. A. Reisman , J. W. Shay , Toxicol. Sci. 2016, 149, 111. 26443840 10.1093/toxsci/kfv213 PMC5013822

[47] D. Xu , L. Chen , X. Chen , Y. Wen , C. Yu , J. Yao , H. Wu , X. Wang , Q. Xia , X. Kong , Cell Death Dis. 2017, 8, e2983. 28796242 10.1038/cddis.2017.386 PMC5596572

[48] A. M. Bataille , J. E. Manautou , Clin. Pharmacol. Ther. 2012, 92, 340. 22871994 10.1038/clpt.2012.110 PMC3704160

[49] E. Ramos‐Tovar , P. Muriel , J. Appl. Toxicol. 2019, 40, 151. 31389060 10.1002/jat.3880

[50] S. M. Shin , J. H. Yang , S. H. Ki , Oxid. Med. Cell Longev. 2013, 2013, 763257. 23766860 10.1155/2013/763257 PMC3665261

[51] W. Tang , Y. F. Jiang , M. Ponnusamy , M. Diallo , World J. Gastroenterol. 2014, 20, 13079. 25278702 10.3748/wjg.v20.i36.13079 PMC4177487

[52] D. Xu , M. Xu , S. Jeong , Y. Qian , H. Wu , Q. Xia , X. Kong , Front. Pharmacol. 2018, 9, 1428. 30670963 10.3389/fphar.2018.01428 PMC6331455

